# Retinal glia in myopia: current understanding and future directions

**DOI:** 10.3389/fcell.2024.1512988

**Published:** 2024-12-20

**Authors:** Pengfan Chen, Jing Ji, Xinyi Chen, Jiali Zhang, Xiangyi Wen, Longqian Liu

**Affiliations:** ^1^ Department of Ophthalmology, Laboratory of Optometry and Vision Sciences, Department of Optometry and Visual Science. West China Hospital, Sichuan University, Chengdu, Sichuan, China; ^2^ West China school of Medicine, Sichuan University, Chengdu, Sichuan, China

**Keywords:** myopia, retina, glia, astrocyte, Müller cells, Microglia

## Abstract

Myopia, a major public health problem, involves axial elongation and thinning of all layers of the eye, including sclera, choroid and retina, which defocuses incoming light and thereby blurs vision. How the various populations of glia in the retina are involved in the disorder is unclear. Astrocytes and Müller cells provide structural support to the retina. Astrogliosis in myopia may influence blood oxygen supply, neuronal function, and axon diameter, which in turn may affect signal conduction. Müller cells act as a sensor of mechanical stretching in myopia and trigger downstream molecular responses. Microglia, for their part, may exhibit a reactive morphology and elevated response to inflammation in myopia. This review assesses current knowledge about how myopia may involve retinal glia, and it explores directions for future research into that question.

## Graphical Abstract

**Figure:**
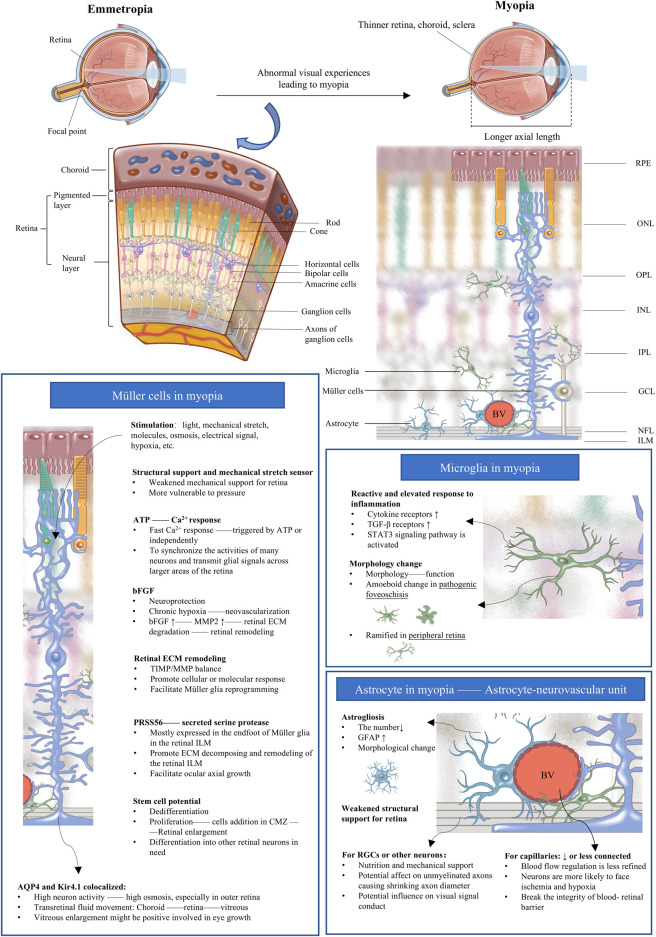
Summary diagram of the potential mechanism of retinal glial cells implicated in myopia. The figure shows the anatomical relationship of the three retinal glial cells including astrocyte, microglia, and Müller cells to other neurons, and summarizes the potential mechanisms by which glial cells respond to myopia.

## 1 Introduction

**FIGURE 1:**
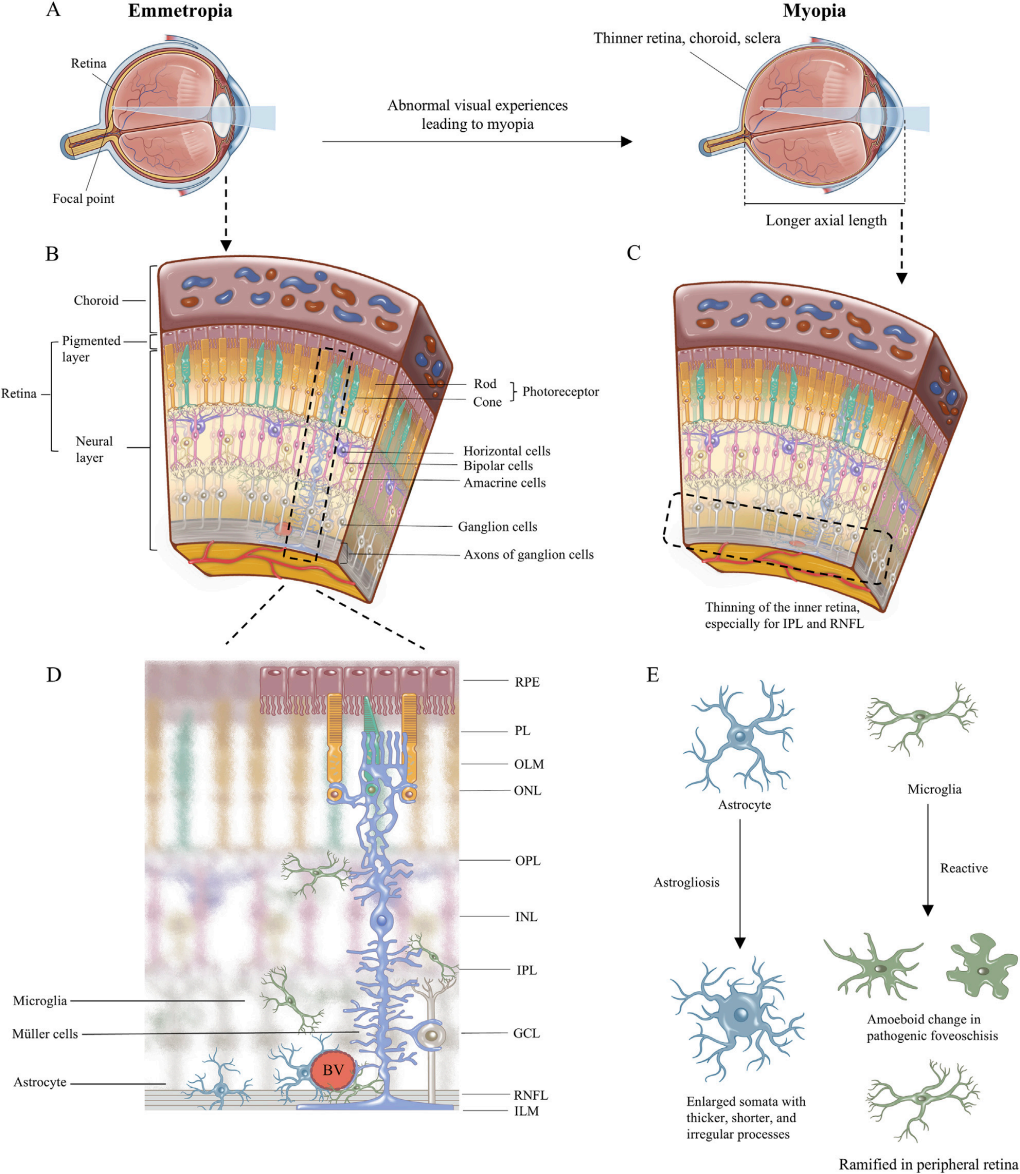
The illustration of myopia change and the retinal cellular arrangement. **(A)** The image focuses on the retina in emmetropia eye, while in myopia eye the image focuses in front of retina because of the longer axial length with thinner retina, choroid, and sclera. **(B)** Retinal cellular arrangement and morphology are shown. **(C)** Schematic diagram of retinal anatomical changes in myopia, with thinning of the inner retina, especially for IPL and RNFL. **(D)**The morphology of the three retinal glial cells including astrocyte, microglia, and Müller cells, and their anatomical relationship are emphatically demonstrated. **(E)** The morphological changes of glial cells in myopia have been reported in the literature. RPE, retinal pigmented epithelium monolayer; PL, photoreceptor layer; OLM, outer limiting membrane; ONL, outer nuclear layer; OPL, outer plexiform layer; INL, inner nuclear layer; IPL, inner plexiform layer; GCL, ganglion cell layer; RNFL, retinal nerve fiber layer; ILM, inner limiting membrane. BV, blood vessels.

The prevalence of myopia continues to increase, such that it is expected to affect nearly half the global population by 2050 (Holden et al., 2016). Uncorrected myopia is one of the most frequent causes of visual impairment (Kido et al., 2024), and its progression can damage the retina, choroid and sclera (Baird et al., 2020). Although myopia is a major public health problem, how it occurs and how it involves different populations of cells in the eye are unclear. Various processes have been proposed to contribute to myopia, such as reduced dopamine signaling (Ji et al., 2022), choroidal thinning and associated ischemia (Ostrin et al., 2023), hypoxia and remodeling of the sclera (Wu et al., 2018), as well as inflammation and oxidative stress (Xu et al., 2023). The retina, as the first part of the eye that encounters abnormal visual signals, may play a central role in myopia by triggering excessive eye growth in response to those signals (Brown et al., 2022; Yao et al., 2020; Pusti et al., 2024; Shu et al., 2023) (Figure 1A).

Retinal glia, which outnumber retinal neurons by at least a factor of 10 (Miao et al., 2023), are likely to be involved in myopia. Here we review our current understanding of how different populations of glia in the retina may be involved in myopia, and we propose future directions for research to deepen that understanding.

## 2 Concise anatomy of the retina and description of myopia

The retina is a sophisticated visual sensory tissue with a laminar structure comprising the following ten layers from outside to inside (Hoon et al., 2014) (Figures 1B, D): retinal pigmented epithelium (RPE) monolayer, photoreceptor layer (PL), outer limiting membrane (OLM); outer nuclear layer (ONL), outer plexiform layer (OPL), inner nuclear layer (INL), inner plexiform layer (IPL), ganglion cell layer (GCL), retinal nerve fiber layer (RNFL), and inner limiting membrane (ILM). Incoming light is sensed by photoreceptors in the ONL, which transmit visual signals directly and via bipolar cells in the INL. Horizontal cells and amacrine cells in the INL fine-tune the visual signal horizontally in the OPL and IPL, respectively. The fine-tuned signals are coordinated in retinal ganglion cells, which send the input to higher visual processing centers in the brain (Hoon et al., 2014; Masland, 2001).

Axial myopia involves substantial lengthening of the ocular axis, enlargement of the vitreous, reduced choroidal blood flow, and decreased choroidal thickness. The inner retina becomes thinner, especially the RNFL and IPL, than all the retinal layers (Lin et al., 2024; Chen CY. et al., 2018; Swiatczak et al., 2019a; Zha et al., 2017; Ablordeppey et al., 2024). Although the onset of myopia remains unclear, ischemia-hypoxia, inflammation, and oxidative stress are recognized the potential pathological responses in myopia (Ostrin et al., 2023; Wu et al., 2018; Xu et al., 2023). Glia is implicated in those stress. Astrogliosis in myopia may influence blood oxygen supply, neuronal function, and axon diameter (Lin et al., 2024; Benavente-Pérez et al., 2010; Hawkins and Davis, 2005; Leng et al., 2018; Swiatczak et al., 2019b). Astrocytes and Müller cells provide structural support in normal retina (Lundkvist et al., 2004; Bringmann et al., 2022). In ocular elongation suffering from mechanical stress in myopia, Müller cells especially may act as a sensor of mechanical stretching (Lindqvist et al., 2010). Their abnormalities lead to fragile structure of retina to cope with intraocular pressure and mechanical forces with fast-growing of eyeball. The downstream molecular responses within Müller cells can transmit glial signals and synchronize the activities of many other neurons (Newman and Zahs, 1997; Newman and Zahs, 1998; Rillich et al., 2009). Müller cells play roles in retinal extracellular matrix remodeling, providing an incipient looser microenvironment for migration of cells, cytokines, or inflammatory factors in myopia (McBrien and Gentle, 2003; Varshney et al., 2015; Long and Huttner, 2019; Liu et al., 2017; Zhou et al., 2023). Microglia may exhibit a reactive morphology and elevated response to inflammation in myopia (Yao et al., 2020). Due to the limited evidence, it is hard to summary the role of glia in the onset of myopia, high myopia or pathological myopia respectively, or whether glia responses promote myopia progression. How each glial cell respond to abnormal visual experiences in myopia will be described in detail here.

## 3 Location, morphology, and function of glia in the retina

Glia, comprising mainly microglia and two types of macroglia called astrocytes and Müller cells (Reichenbach and Bringmann, 2020), reside primarily in the inner retina. Microglia sense inflammatory signals and strive to maintain homeostasis in the retina. Astrocytes modulate the activity of neurons and retinal ganglion cells in order to support the transfer of visual signals to higher vision centers in the brain. Müller cells respond to ocular stretching and ocular fluid regulation, thereby helping to regulate and refine visual signals to the outer ocular layers (Figure 1D).

### 3.1 Astrocytes

Astrocytes migrate into the retina along with its vasculature and concentrate within the RNFL. The number and location of astrocytes in the retina strongly correlate with the number and location of blood vessels and nerve fibers there (Vecino et al., 2016). The parafoveal and foveal regions feature four layers of vasculature and two layers of astrocytes, one of which is a superficial layer close to the ILM, while the other is a deeper layer close to the GCL. The peripapillary region contains three layers of vasculature and one layer of astrocytes. The peripheral region features two layers of vasculature and one layer of astrocytes (Lin et al., 2024; Lin et al., 2022).

The cell bodies of astrocytes aggregate in the RNFL, and their processes reach axons in the retinal ganglion cells as well as vasculature and other glia within the RNFL (Holden et al., 2024; Cameron et al., 2024). Astrocytes can be identified based on their expression of glial fibrillary acidic protein (GFAP), the primary type of intermediate filament in the vertebrate nervous system (Mokhtar et al., 2024). This and other intermediate filaments provide structural and mechanical support to maintain cellular morphology (Hol and Pekny, 2015), while also coordinating mechanical sensing, transduction, signaling, motility, and inflammatory responses (Ridge et al., 2022).

### 3.2 Müller cells

From the somata of Müller cells in the INL radiate two stem processes in opposite directions, spanning from the ILM to the ONL, which is nearly the entire thickness of the retina. The inner stem process terminates in a funnel-shaped endfoot. Lateral processes extend into the plexiform layers to form sheaths around synapses, while also extending into the nuclear layers to embed in neuronal perikarya.

Their unique morphology allows Müller cells to interact with all neurons of the retina (Devoldere et al., 2019) and modulate synaptic activity (Reichenbach and Bringmann, 2020). Müller cells play a crucial role in retinogenesis, transmit various molecules between different retinal cells (Too and Simunovic, 2021) and participate in the establishment and maturation of the blood–retinal barrier (Biswas et al., 2024). They support neurons by releasing trophic factors and neurotransmitters as well as by regulating extracellular ion homeostasis. Like astrocytes, Müller cells can also be identified based on their expression of GFAP (Mokhtar et al., 2024), and both types of macroglia provide mechanical support to the retina through strands of microtubules and intermediate filaments such as GFAP and vimentin (Lundkvist et al., 2004; Bringmann et al., 2022). In addition to playing this supporting role in transmission of visual signals, Müller cells can themselves play the main role of light detection and phototransduction (Goldman, 2014; Franze et al., 2007). Moreover, Müller cells contribute to the visual cycle of cone cells by phagocytosing their outer segments to promote their turnover and biosynthesis (Reichenbach and Bringmann, 2020; Karlen et al., 2020).

### 3.3 Microglia

Microglia are the primary resident innate immune cells of the central nervous system. They contribute to programmed cell death, neurogenesis, vascular development, and refinement of synapses and neuronal circuits. In the healthy mature retina, microglia make up a stable and highly ordered network of ramified cells that are thought to carry out constitutive maintenance functions as well as regulate neuronal activity and synaptic integrity. They are usually distributed horizontally in the synaptic OPL, IPL and nuclear GCL of the retina, as well as around blood vessels (Silverman and Wong, 2018). Microglia in the central nervous system derive from hematopoietic progenitors in the extraembryonic yolk sac (Ginhoux and Prinz, 2015). When microglia enter the developing retina, amoeboid cells that express markers of microglia or macrophages emerge in the vitreous and on the vitreal surface of the embryonic retina, near the optic nerve, and in the peripheral retina. Then they simultaneously proliferate and migrate radially and horizontally to occupy the entire retina. Their morphology becomes polarized and their processes ramify (Silverman and Wong, 2018).

## 4 Myopia disproportionately affects the inner retina, where most glia cells localize

Excessive eye growth in myopia leads to thinning that is more severe in the inner retina than in other retinal layers (Lin et al., 2024; Chen CY. et al., 2018; Swiatczak et al., 2019a; Zha et al., 2017; Ablordeppey et al., 2024). Experiments in different systems suggest that the thinning affects the RNFL and IPL (Lin et al., 2024; Swiatczak et al., 2019b) (Figure 1C). Experiments in a marmoset model of myopic foveoschisis suggest that myopia involves not only mechanical stretching of the retina but also gliosis (Sin et al., 2023). In a marmoset model, myopia did not obviously affect photoreceptors in the outer retina (Sin et al., 2023). Electroretinographic studies of marmoset models support the idea that myopia affects primarily the inner retina and not the outer retina (Ablordeppey et al., 2024), and that changes of electrophysiology in bipolar, retinal ganglion, amacrine and glial cells may precede pathology of the inner retina (Thompson et al., 2011; Kumar et al., 2022; Viswanathan et al., 1999; Viswanathan et al., 2001; Machida et al., 2008).

The observation that myopia involves pathology primarily in the inner retina, where most glia localize, prompts the question, “How do retinal glia contribute to myopia?”

## 5 Astrocytes in myopia

### 5.1 Astrogliosis

**FIGURE 2:**
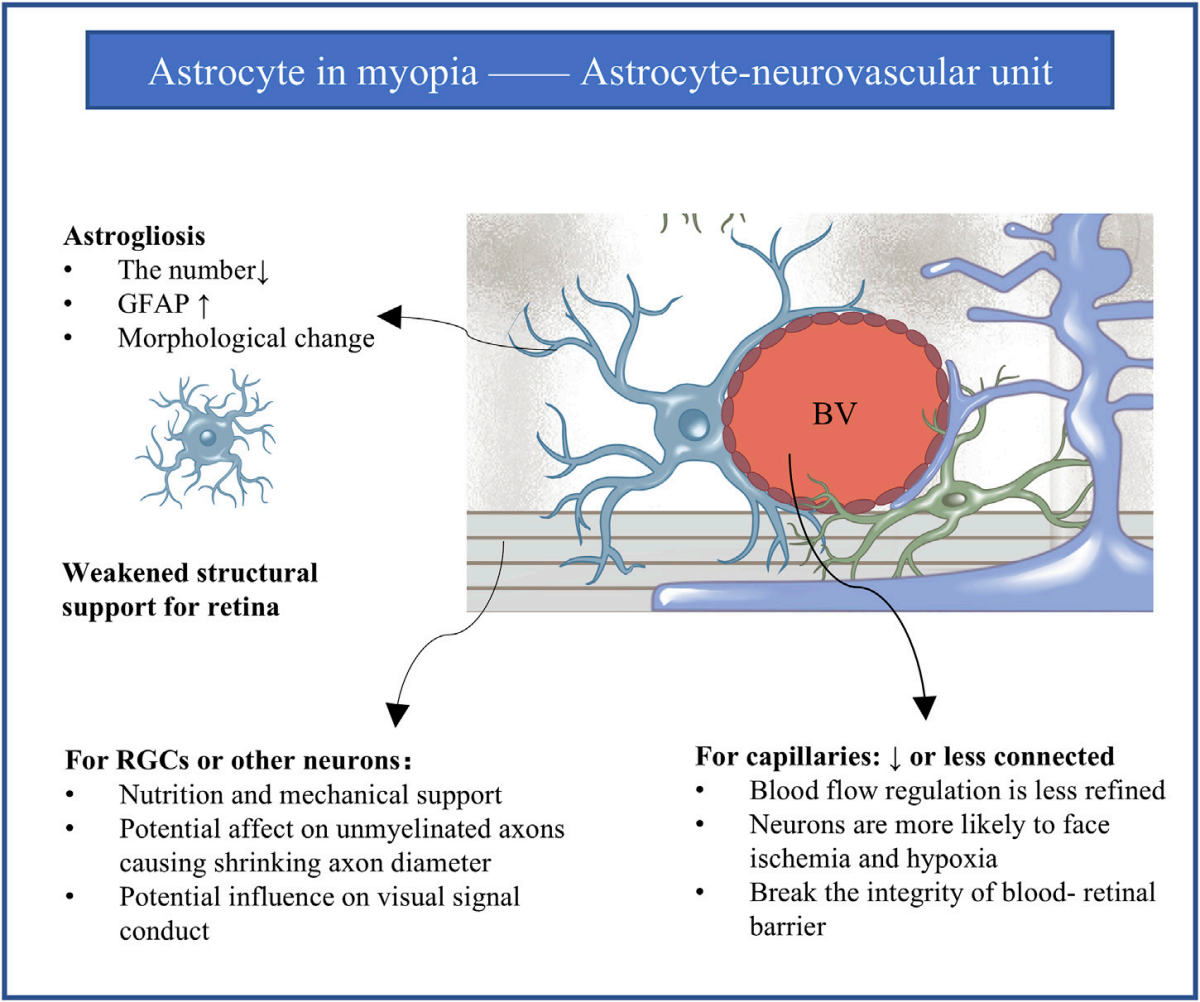
The astrocyte-neurovascular unit and influence of astrogliosis in myopia. Every astrocyte in the retina contacts at least one blood vessel and at least one neuronal element to mediate the integration between blood vessels and neurons. Astrogliosis is implicated in myopia progression. Gliosis for the intermediate astrocyte reasonably leads to abnormalities in blood vessels and neurons. GFAP, glial fibrillary acidic protein; RGCs, retinal ganglion cells; BV, blood vessel.

Quiescent astrocytes are activated respond to adverse factors, leading to changes in morphology, gene expression, and functions, known as “astrogliosis” (Miao et al., 2023). One hallmark of astrogliosis is upregulation of GFAP. Such gliosis is a double-edged sword: it can protect retinal ganglion cells from further injury, yet it can also promote their death (Reichenbach and Bringmann, 2020). Myopia is likely to involve astrogliosis (Figure 2). In a marmoset model of myopia, myopic eyes showed a smaller number of astrocytes yet higher expression of GFAP, consistent with astrogliosis (Lin et al., 2024). Astrocytes in the radial peripapillary capillary layer in fovea showed enlarged somata with thicker, shorter, and irregular processes (Figure 1E). Astrocytes in the superficial vascular plexus in fovea, peripapillary, and peripheral retina also showed hypertrophy and hyperdense processes (Lin et al., 2024; Lin et al., 2022). These pathological changes worsened with time. Similar results were observed in a mouse model of myopia (Zhang et al., 2022). The horizontal GFAP area in the myopic eye was thicker than in the control eye, and the myopic eye showed fibers deep in the IPL, which was much thicker than the control eye (Zhang et al., 2022). In the control eye, GFAP expression was restricted to the inner side of retinal ganglion cells. In a rhesus macaque model of myopic foveoschisis, gliosis of macroglia was found at the central macula, where foveoschisis was most prominent and foveal pit morphology most severely disrupted, yet photoreceptors showed only minor disruption (Sin et al., 2023).

These studies suggest that in myopia, astrocytes in foveal and peripheral retina show astrogliosis involving altered morphology and upregulation of GFAP. The formation of thin, long filaments running in one direction along the deeper vasculature of the RNFL, especially in peripapillary and peripheral retina, is probably the result of mechanical stretching. Myopia likely interacts with age to affect retinal astrocytes, given that aging is associated with decreased astrocyte density and astrogliosis (Mansour et al., 2008; Ramírez et al., 2001; Madigan et al., 1994; Cavallotti et al., 2003).

So far, direct evidence linking astrogliosis to myopia in humans is lacking, although glia cells have been detected in ILM from patients with myopic foveoschisis (Vogt et al., 2020; Yokota et al., 2018; Chen L. et al., 2018). The ILM is a specialized basement membrane located at the border between the vitreous body and retinal neuroepithelium. One possibility is that the ILM stiffens through upregulation of GFAP, reactive gliosis, abnormal collagen formation and long insertions of process of glia cells. On the other hand, such stiffening of the ILM can inhibit gliosis (Halfter et al., 2006). Stiffening of the ILM may also generate severe traction force, putting significant mechanical stress onto foveal layers and causing foveoschisis lesions. Whatever the detailed mechanism(s), the development of myopia in humans involves transfer or reorganization of astrocytes (Lin et al., 2024), because ILM from myopic individuals shows increased astrocyte density (Chen L. et al., 2018). However, astrocyte density was decreased in all retinal regions of a marmoset model of myopia (Lin et al., 2024). Further research should explore the potential contribution of gliosis in myopia.

### 5.2 Astrogliosis and the astrocyte-neurovascular unit

Every astrocyte in the retina contacts at least one blood vessel and at least one neuronal element such as the soma or axon of retinal ganglion cells, allowing them to guide vascular development (O’Sullivan et al., 2017) and mediate the integration between blood vessels and neurons to create the so-called astrocyte-neurovascular unit (Hawkins and Davis, 2005) (Figure 2). This unit appears to be crucial for retinal structure (Hawkins and Davis, 2005; Vecino et al., 2016), modulation of vascular tone, regulation of blood flow and integrity of the blood-retinal barrier (Holden et al., 2023; Sapieha, 2012), as well as metabolism, neuronal turnover and neurotransmitter homeostasis (Hawkins and Davis, 2005; Vecino et al., 2016; Sapieha, 2012). It seems reasonable to assume that astrogliosis in myopia can alter the physiology and processing activity of retinal ganglion cells and other neurons, leading to abnormal retinal vascularization and weakened structural support.

Consistent with this idea, myopic eyes in humans and animal models show decreased blood supply to the retina and simultaneous loss of astrocytes and their associated capillaries across the retina, together with slower blood flow in the central retinal artery (Benavente-Pérez et al., 2010; Leng et al., 2018), narrowing of retinal vessels (Leng et al., 2018), lower capillary density (Holden et al., 2016), larger avascular zones in the fovea (Gołębiewska et al., 2019; Wu et al., 2021), and loss of vascular branching in the periphery and peripapillary regions (Bucher et al., 2013; Cheng et al., 2021). Vascular branching in the fovea may increase as a compensatory mechanism, at least in a marmoset model of myopia (Lin et al., 2022). All these indicators of vascular reorganization suggest hypoxia in the myopic periphery (Shih et al., 1993). Supporting that, four genes were shared as reported between hypoxic astrocytes and human myopia, which are *GRIA4*, *RP2*, *CNGB3*, and *ADAMTS10*. *GRIA4* is expressed in the cone ON bipolar cells and is responsible for the common refractive error. *RP2* and *CNGB3* are expressed in cones and rods and are associated with syndromic myopia. *ADAMTS10* is expressed in the sclera (Zhang et al., 2022).

In addition to affecting blood flow, astrogliosis in the astrocyte-neurovascular unit may also affect neuronal transmission. Astrogliosis has been linked to demyelination, compensatory remyelination, and axon loss in the developing and diseased brain (van Deijk et al., 2017; Mi et al., 2023; Wan et al., 2022; Zheng et al., 2021). Normally, axons of retinal ganglion cells at the optic nerve head within the retina are unmyelinated, while the proportion of myelinated retinal ganglion cells increases as one moves toward the brain (Young et al., 2013). In chickens, in which intraocular myelination of ganglion cell axons is normal, myopia reduced the thickness of the RNFL by about 14% and the thickness of unmyelinated axons by about 29%, while also reducing the total number of myelinated axons (Swiatczak et al., 2019b). The velocity and fidelity of visual signal conduction depend mainly on myelin sheath length and axon diameter (Nabel et al., 2024): for example, larger axon diameter translates to faster signal conduction (Olausson et al., 2024; Horowitz et al., 2015; Ritchie, 1982). Thus, demyelination and shrinking axon diameter may slow visual signal conduction and render the neurons more susceptible to hypoxic injury, especially in the presence of myopic pathology such as myopia-associated astrogliosis.

These considerations raise the question: what happens to unmyelinated retinal axons of humans or mice in the presence of myopia? Chronically abnormal visual stimuli may directly influence the axons without myelin, even though glia may play a compensatory role like myelin.

Differential vulnerability of the astrocyte-neurovascular unit in different parts of the eye may help explain why the excessive growth in myopia disproportionately affects the peripheral retina. This region and the optic nerve head, which are less stiff than the mid-retina (Franze et al., 2011), contain astrocytes with compressed morphology that probably contact fewer unique blood vessels. As a result, astrocytes in these regions cannot regulate blood flow as effectively as in the mid-retina, especially in the presence of myopic injury (Holden et al., 2023).

## 6 Müller cells in myopia

**FIGURE 3:**
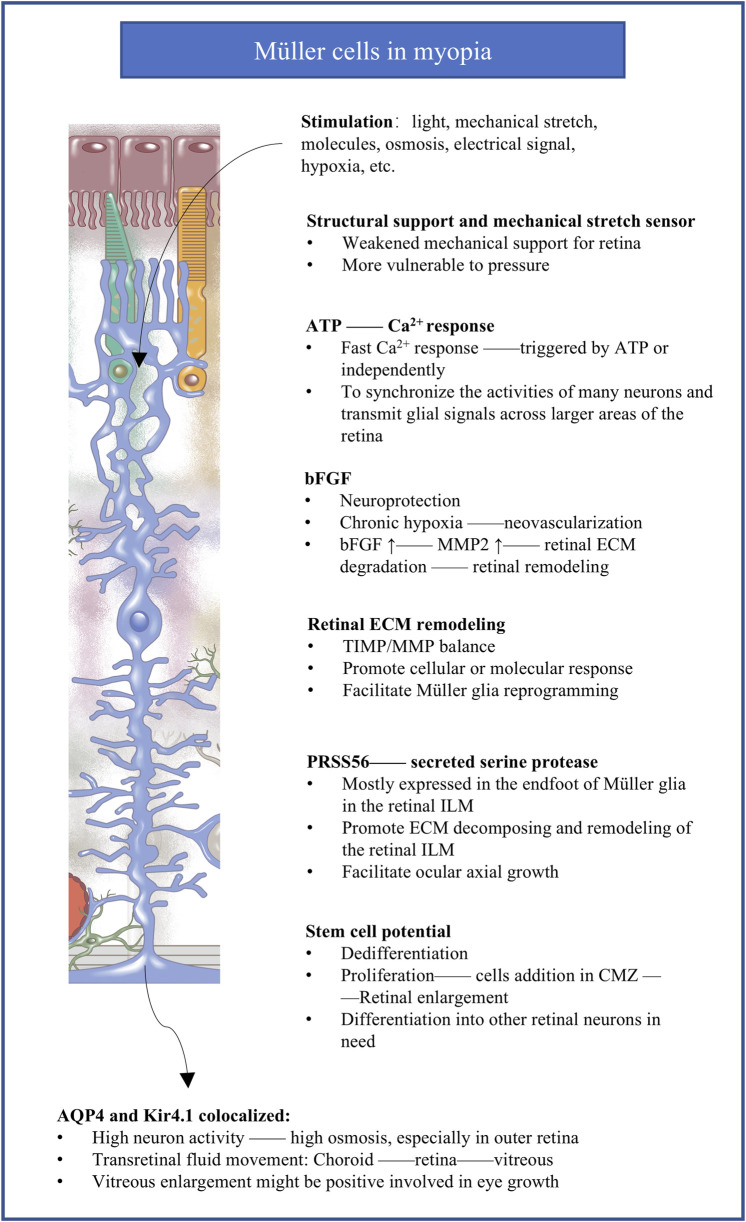
The potential molecular responses of Müller cells implicated in myopia. The figure summarizes the potential mechanisms of Müller cells responding to abnormal visual stimuli in myopia. bFGF, basic fibroblast growth factor; MMP, matrix metalloprotease; ECM, extracellular matrix; TIMP, tissue inhibitors of metalloproteases; ILM, inner limiting membrane; PRSS56, secreted serine protease 56; CMZ, circumferential marginal zone; AQP4, aquaporin 4; Kir4.1, inward-rectifying K^+^ channel from the 4.1 subfamily.

The literature suggests numerous mechanisms through which Müller cells may contribute to myopia (Figure 3).

### 6.1 Müller cells and ATP production and degradation

Inhibiting adenosine receptors, which are activated by the adenosine from ATP breakdown (Idzko et al., 2014), inhibits myopia in animal models (Hung et al., 2018; Smith et al., 2021; Liu et al., 2020). The vitreous humor from myopic individuals with complications contains elevated level of uric acid (Tang et al., 2023), the end-product of ATP breakdown. These observations lead to the proposal that progression of myopia involve elevation in extracellular ATP and its degradation into adenosine and uric acid. This is consistent with the activity of extracellular ATP as a danger signal (Resta et al., 2007) that can activate P2X or P2Y receptors to stimulate inflammation and other pathological signaling cascades (Linden et al., 2019).

Indirect evidence implicates Müller cells in the release of ATP outside cells in myopia. These cells can release ATP in response to light, osmotic or mechanical stress, as well as following activation of purinergic, dopaminergic and glutamatergic receptors (Newman, 2003; Newman, 2001a; Newman, 2001b). Activation of purinergic receptors has been shown to induce gliosis of Müller cells yet also their hypertrophy and proliferation (Reichenbach and Bringmann, 2016). Exposing Müller cells to ATP, light, electrical or mechanical stimulation can trigger them to release Ca^2+^ from intracellular stores, which synchronize the activities of many neurons and transmit glial signals across larger areas of the retina (Newman and Zahs, 1997; Newman and Zahs, 1998; Rillich et al., 2009).

### 6.2 Müller cells and hypoxia

A growing body of literature propose that hypoxia modulates myopia development (Wu et al., 2018). The longer axial length in myopia is associated with thinning of the choroids (Ostrin et al., 2023; Shen et al., 2024), which may in turn lead to less blood perfusion in retina. This creates a hypoxic environment for photoreceptors in the outer retina, which is one of the most metabolically demanding tissues and which relies mainly on perfusion of choroid tissue (Alm and Bill, 1973). Chronic hypoxia appears to upregulate secretion of basic fibroblast growth factor (bFGF) by Müller cells, which stimulates the proliferation of retinal vascular endothelial cells to drive neovascularization (Peng et al., 1998; Soubrane et al., 1994; Bringmann et al., 2009; Bringmann et al., 2006).

### 6.3 Müller cells and mechanical stretching

Myopia involves excessive elongation of the eye axis, which inevitably involves mechanical stretching. Müller cells can sense such stretching and other subtle alterations because of their unique transretinal morphology with long, branched processes. Stretching induces in Müller cells rapid, transient increases in intracellular Ca^2+^ as well as slower, longer-lasting changes in gene expression (Lindqvist et al., 2010), and it triggers responses involving transcriptional factors and molecules involved in ocular axial growth (Mammoto et al., 2012; Wang et al., 2013a). Among these changes in expression is initial upregulation of bFGF (Lindqvist et al., 2010; Bringmann et al., 2009; Cao et al., 1997; Wen et al., 1995; Geller et al., 2001; Fu et al., 2015), which in turn leads to upregulation of matrix metalloprotease (MMP)-2 (Lindqvist et al., 2010; Wang et al., 2013b; Bikfalvi et al., 1997; Limb et al., 2002), which cleaves proteins of the extracellular matrix (Zhang et al., 2010; Page-McCaw et al., 2007; Rosenberg, 2009) and can make the retina less stiff, thereby protecting the retina at an early phase from damage induced by stretching. In the presence of chronic mechanical stress (Mao et al., 2006), levels of bFGF and MMP-2 decrease, which may also protect the eye by strengthening the retina and sclera from serious damage. While the initial upregulation and subsequent downregulation may serve to protect the eye, they may not so benefit for maintaining emmetropia.

### 6.4 Müller cells and remodeling of the extracellular matrix

Müller cells may influence ocular growth not only by secreting MMPs that degrade the extracellular matrix (ECM), but also by secreting MMP inhibitors called tissue inhibitors of metalloproteases (TIMPs) (Campbell et al., 2019). In fact, retinal damage induces Müller cells to upregulate their secretion of TIMP2 and downregulate their secretion of matrix-degrading gelatinase (Campbell et al., 2019). In these ways, Müller cells may influence the balance between degradation and formation of the matrix (McBrien and Gentle, 2003), which in turn may loosen or stiffen the retina and alter its shape (Varshney et al., 2015; Long and Huttner, 2019). Individuals with high myopia show upregulation of MMPs and TIMPs in the aqueous humor (Liu et al., 2017), while experiments in a rat model have suggested that knockout of TIMP4 can contribute to high myopia by reducing collagen content in the sclera and retina (Zhou et al., 2023). Future research is needed to clarify in detail how the extracellular matrix in the retina is altered in myopia and what mechanisms drive those alterations. Such work should consider the apparently two-way communication between Müller cells and the extracellular matrix: the cells secrete MMPs and TIMPs to affect the matrix, while remodeling of the matrix influences the (de)differentiation and proliferation of Müller cells (Wan et al., 2012; Kaur et al., 2018; Naitoh et al., 2017).

Few attentions have been paid to how myopia involves extracellular matrix in the retina rather than in the sclera. The matrix is less abundant in the retina than the sclera, so responses of matrix deposition and remodeling in the retina may be less effective at resulting in ocular elongation. Instead, it prefers to providing incipient looser retinal microenvironment for the molecules, glia, and neurons to respond to abnormal visual experiences in myopia. Anomalous matrix deposition can stiffen the retina and trigger inflammation, leading to scar formation and fibrosis (Melrose et al., 2021; Eamegdool et al., 2020; Ghorbani and Yong, 2021); matrix degradation, conversely, can protect the retina from mechanical stress.

### 6.5 Müller cells and ILM remodeling

The endfoot of Müller cells secretes serine protease 56 (PRSS56) into the ILM of the retina (Uechi et al., 2014), and this protein, as well as the transmembrane glycoprotein membrane frizzled-related protein (MFRP), may help drive excessive growth of the ocular axis. Mutations in the genes encoding either protein lead to a shorter ocular axis in humans and mice (Paylakhi et al., 2018; Velez et al., 2017; Li et al., 2024), and loss of either protein reverses the ability of mutations in the gene encoding the interphotoreceptor retinoid-binding protein (IRBP) to drive excessive growth of the ocular axis (Koli et al., 2021; Wisard et al., 2011). Given that MFRP is expressed predominantly in the retinal pigment epithelium and that IRBP is expressed primarily in the interphotoreceptor matrix between the retinal pigment epithelium and photoreceptors, these proteins, together with PRSS56, may mediate the ability of Müller cells to influence the retinal pigment epithelium and transmit information during ocular growth.

The PRSS56 secreted by Müller cells may help degrade the extracellular matrix and remodel the ILM, which in turn may facilitate ocular axial growth (Halfter et al., 2006). Providing mechanical support to the ILM or inner retina has been shown to reduce Müller cell gliosis and protect neurons in an *in vitro* model (Taylor et al., 2014). These results, suggest a therapeutic strategy against myopia.

### 6.6 Müller cells and transretinal fluid movement

Why the vitreous becomes enlarged in myopia remains a mystery, though numerous studies seem to suggest that the enlargement is the consequence of axial elongation rather than the passive result of increased vitreous or ocular volume (Liang et al., 2004; Crewther et al., 2006). An unexplored possibility is that the enlargement occurs when abnormal visual experiences lead to neural activity that alters ion concentrations and osmotic potential, causing transretinal fluid movement into the vitreous. Müller cells are well-suited to facilitate transretinal fluid movement because of their morphology spanning the retina (Nagelhus et al., 1999; Goodyear et al., 2008; Jo et al., 2015; Bringmann et al., 2004). Osmoregulation is crucial for the retina because its high energy demand and metabolic turnover require efficient systems for preventing water accumulation (Nagelhus et al., 1999; Jo et al., 2015; Moseley et al., 1984; Rathore et al., 2024; Goodyear et al., 2010).

The rate of net fluid transfer between the vitreous and choroid (Crewther, 2000) as well as alterations in choriocapillaris permeability (Pendrak et al., 2000; Hirata and Negi, 1998) may contribute to the redistribution of water in myopia. In a chicken model of myopia, rapid axial elongation and movement of fluid into the vitreous cavity were associated with upregulation of aquaporin 4 in the nerve fiber layer (Goodyear et al., 2010). In that model, upregulation of the inward-rectifying K^+^ channel from the 4.1 subfamily (Kir4.1) appeared to limit axial elongation. These considerations are consistent with the central role of Müller cells in myopia, because the endfeet of these cells express aquaporin 4 at interfaces with retinal capillaries, the vitreoretinal border and synapses in the plexiform layers to facilitate retinal signal transduction (Goodyear et al., 2008; Nagelhus et al., 1998; Iandiev et al., 2007). The endfeet of Müller cells facing the vitreous and blood vessels of the mammalian retina co-express aquaporin 4 and Kir4.1 (Nagelhus and Ottersen, 2013), and these regions act as K^+^ sinks to limit concentrations of K^+^ in the extracellular space around active neurons (Newman, 1993). One possibility is that under normal conditions, aquaporin 4 in the inner retina supports rapid fluid flow across the retina into the vitreous, and it cooperates with ion cotransporters in the retinal pigment epithelium to transport fluid out of the retina and into the choroid. The abnormal visual signaling in myopia may disturb osmotic homeostasis and alter aquaporin 4 expression in Müller cells, leading to excess fluid movement and deposition in the vitreous chamber and, potentially, reduced fluid outflow into the choroid, ultimately leading to ocular enlargement (Goodyear et al., 2009). Consistent with this hypothesis, a chicken model of myopia showed substantial increases in levels of K^+^, Na^+^ and Cl^−^ in the outer retina as well as increases of Na^+^ and Cl^−^ in the inner retina (Liang et al., 2004; Crewther et al., 2006).

One mechanism proposed for myopia is that as photoreceptors sense blurred images, the concentration of K^+^ increases, and choroid vessels respond quickly to the increase in osmotic pressure in the retina, leading to excessive fluid accumulation. The endfeet of Müller cells in the outer retina sense the accumulation and attempt to compensate for it by upregulating aquaporin 4 and downregulating Kir4.1. The higher extracellular concentration of K^+^ in myopia may lead to neuronal excitability, which should be explored in future research.

### 6.7 Müller cells and reprogramming

Müller cells can dedifferentiate into progenitor cells and they can differentiate into a damaged cell type (Huang et al., 2024; Cha et al., 2023; Fischer and Reh, 2001; Bernardos et al., 2007; Fischer and Bongini, 2010; Kubrusly et al., 2005; Loiola and Ventura, 2011) under the influence of cellular and environmental factors (Fischer and Reh, 2001). Progenitors of Müller cells form a circumferential marginal zone (CMZ) that lines the periphery of the retina. Normally the retina provides signals that suppress proliferation of progenitors in the CMZ (Fischer and Reh, 2001), but induction of myopia stimulates proliferation of those progenitors which is associated with eye growth (Fischer, 2011). Glucagonergic amacrine cells with massive neurites cluster around the progenitors and may contribute to myopia progression (Fischer and Reh, 2000). These new additional cells derived from proliferation of progenitors of Müller cells may not only enlarge retina, but also provide more abilities to differentiate into other functional neurons supporting the retina.

## 7 Microglia in myopia

**FIGURE 4:**
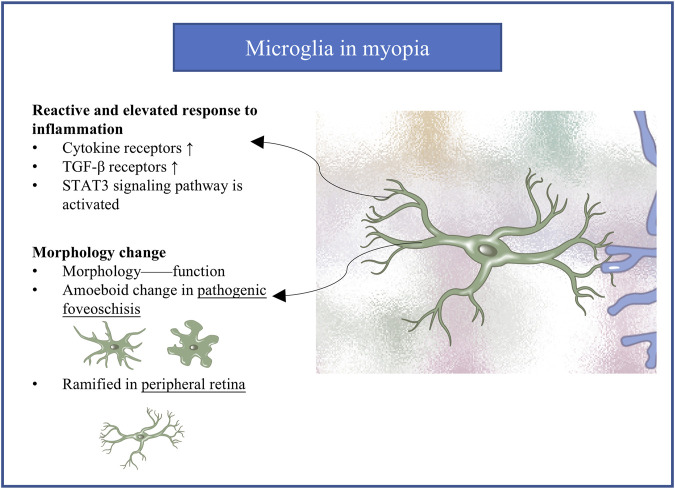
The molecular and morphological change of microglia in myopia. Microglia exhibits reactive and elevated response to inflammation. Amoeboid morphological change in pathogenic foveoschisis. TGF-β, transforming growth factor-β.

Quite little is known about the role of microglia in the progression of myopia (Figure 4). A single-cell RNA sequencing research performed on mouse discovered that microglia activity was increased in high myopic retinas (Yao et al., 2020). *Il1a*, *Il6ra*, *Il21r (interleukin, IL)*, *Tgfbr1*, and *Tgfbr2 (transforming growth factor-β, Tgfb)* and downstream transcriptional regulators (*Stat3*, *Nfkbr1*, and *Nfkbr2*) were found significantly increased in the microglia of highly myopic eyes. It indicates cytokine receptors rather than cytokines, and TGF-β receptors were significantly elevated in microglia which highlight the enhanced responses of highly myopic eyes to proinflammatory environment and the growth-promoting states involved in high myopia progression. STAT3 signaling pathway was activated in highly myopic microglia, exhibiting an aging or neuroinflammation profile. Meanwhile, genes enriched for cell activation, cell migration, and cellular responses to stress were also upregulated in highly myopic microglia (Yao et al., 2020; Lin et al., 2023; Burton et al., 2013). In animal models of myopia, activated microglia in the IPL showed shorter, thicker processes differing from the long, thin, highly branched processes in control retinas (Ritchey et al., 2012; Fischer et al., 2010). In a primate model of pathologic myopic foveoschisis, activated microglia in the fovea showed amoeboid rather than normal dendritic morphology (Sin et al., 2023). Microglia in the peripheral retina, however, showed normal dendritic morphology, and photoreceptors in the retina did not show obvious alterations (Figure 1E). Further research is needed to expand our knowledge of microglia in myopia.

## 8 Future directions

Research is just beginning to elucidate how the various types of glia in the retina adapt to sensing of blurred images in myopia. While neurons have been a traditional focus of ocular research, they are outnumbered by glia, which play vital roles in modulating neuronal processing of visual signals and in regulating eye growth. Our review has identified several areas where future research should deepen and broaden our understanding of how myopia occurs and progresses, thereby identifying potential targets for myopia management.

Studies should attempt to explain the thinning of the inner retina, especially the RNFL and IPL, in myopia. Potential causes include demyelination of ganglion cells and shrinking of their axon diameters, based on results from a chicken model of myopia, in which intraocular ganglion cell axons are myelinated (Swiatczak et al., 2019b). Whether the same is true in humans or mice, in which ganglion cell axons are not myelinated, remains to be seen. Axon damage may contribute to the blurring of images in myopia by compromising the speed and fidelity of visual signal processing, which requires further investigation.

Future studies, especially those *in vivo,* should explore whether and how mechanical stretching contributes to myopia progression, and whether Müller cells are involved. The fact that Müller cells penetrate nearly all retinal layers make them well-suited to sensing mechanical stresses (Lindqvist et al., 2010). In addition to ocular elongation, myopia involves retinal enlargement, and research should explore the potential contribution of Müller cells here as well. The retina is likely to enlarge though a process more complicated than simple stretching, because retinal thinning occurs primarily in the inner retina, not across all retinal layers. Secretion of MMPs and TIMPs by Müller cells and the reprogramming of these cells may remodel the extracellular matrix of the retina to facilitate enlargement (Wan et al., 2012; Kaur et al., 2018; Naitoh et al., 2017).

Like the retina itself, the vitreous also enlarges in myopia, and this has traditionally been regarded as an automatic “byproduct” of ocular elongation and therefore neglected in the literature. However, studies suggest that osmotic changes due to accumulation of extracellular K^+^ in the myopic eye may lead to transretinal fluid movement that is mediated by aquaporin 4 on Müller cells and that leads to vitreous enlargement. This potential mechanism should be explored in future work, which may also help to explain why the choroid thins in myopia.

Studies are urgently needed into the potential role of retinal microglia in myopia, a topic that has been sorely neglected in the literature. How microglia respond to abnormal visual experiences and the retinal microenvironment in myopia remains unknown. An obvious line of investigation to explore is the involvement of a pro-inflammatory environment, which is known to activate microglia (Yao et al., 2020) and thereby alter their morphology and behavior (Ritchey et al., 2012; Fischer et al., 2010). This and other lines of investigation need to examine whether and how microglia contribute to myopia onset and progression.

Ultimately, a major goal in elucidating the roles of retinal glia in myopia is to identify therapeutic targets. To our known, there is not clinic trails or applications targeting retinal glia cells. The standard strategy for controlling excessive axial elongation is to reinforce the posterior sclera with various materials (Ye et al., 2024; Zhong et al., 2024). Another possibility is to reinforce the inner retina or ILM in order to inhibit Müller gliosis (Taylor et al., 2014). This as well as other mechanical and pharmacological approaches to modulating retinal glia should be explored for controlling myopia progression.
